# Study on Synthesis and Regulation of PPVI and PPVII in *Paris polyphylla* with UV

**DOI:** 10.3390/metabo14080427

**Published:** 2024-08-02

**Authors:** Dongjie Geng, Yiqun Sun, Shouzan Liu, Wen Chen, Fei Gao, Yan Bai, Shaobo Zhang

**Affiliations:** 1 Zhejiang Provincial Key Laboratory of Resources Protection and Innovation of Traditional Chinese Medicine, Hangzhou 311300, China; gengdongjie@stu.zafu.edu.cn (D.G.); chenwen012024@163.com (W.C.); gfeitcm@zafu.edu.cn (F.G.); 2 Tea Research Institute, Chinese Academy of Agricultural Sciences, Hangzhou 311300, China; 3 College of Food and Health, Zhejiang Agriculture and Forestry University, Hangzhou 311300, China; 4 State Key Laboratory of Subtropical Silviculture, Zhejiang Agriculture and Forestry University, Hangzhou 311300, China; szliu@zafu.edu.cn; 5 Chun’an County Forestry Bureau, Chun’an 311330, China; yqunsun@163.com

**Keywords:** UV light, saponin content, saponin synthase, Chinese traditional medicine, cancer

## Abstract

*Paris polyphylla* Smith var. *Chinensis* (Franch.) Hara is a medicinal plant that belongs to the Liliaceae family. Its main components are parissaponins, which have excellent medicinal effects such as anti-inflammatory, anti-tumor, etc. Improving the quality of parissaponins through artificial directional regulation has emerged as a practice to meet medical demand and is a new research hotspot. In this paper, *P. polyphylla* plants were treated with UVA, UVB, and UVC, and the contents of PolyPhyllin VI (PPVI) and PolyPhyllin VII (PPVII), saponin synthase (squalene synthase, SS; cycloartenol synthase, CAS; cytochrome P450, CYP450; and glycosyl transferases, GT) activity, MDA, and the photosynthetic pigment indexes were measured and analyzed. The results showed that PPVII content increased by 32.43% with UVC treatment after 4 h (3.43 mg/g), but the PPVI and PPVII contents in the other groups decreased compared with CK (control group) and they did not return to the original level after 4 h. SS, CAS, CYP450, and GT synthases were activated in varying degrees via UV treatment and increased, respectively, by 22.93%, 10.83%, 20.15%, and 25.98%. Among them, GT, as the last of the synthetases, had a shorter response time to UVB (30 min) and UVC (15 min); the difference was sensible compared with CK. Moreover, UV had a stressing effect and promoted the rapid accumulation of MDA content (increased 17.66%, 34.53%, and 9.65%) and carotenoid (increased 7.58, 5.60, and 7.76 times) within 4 h compared to CK. UVB and UVC radiation visibly improved chlorophyll a content (42.56% and 35.45%), but UVA did not, and the change in chlorophyll b content showed no overt statistical difference. In addition, PPVI and PPVII were negatively correlated with SS, CAS, carotenoids, and MDA (*p* < 0.05) and positively correlated with CYP450, GT, and chlorophyll a (*p* < 0.05). This study provides a theoretical basis for using UV light to regulate secondary metabolism in *P. polyphylla*, which is of great value for production management.

## 1. Introduction

*Paris polyphylla* Smith var. *Chinensis* (Franch.) Hara belongs to the Liliaceae family [1]. The dry root tubers (Paridis Rhizoma) have long been used as traditional drug sources for Chinese medicinal materials; they have a long history and are recorded in the Pharmacopoeia of the People’s Republic of China. *P. polyphylla* root tubers have a bitter taste and slightly cold nature, and can clear away heat and toxic materials, subside swelling, relieve pain, and calm a frightened person; as such, they are commonly used in folk medicine to treat convulsions, snakebites, and furunculosis [2,3] and are an important component of Yunnan-Bai-yao, Gong-xue-ning, and other drugs [4]. The main active component in root tubers is steroidal saponin [5]. Among the steroidal saponins, PPVI (PolyPhyllin VI) and PPVII (PolyPhyllin VII) are the pannosaponins with the most significant pharmacological activities [6]. Several modern medical studies have shown that both PPVI and PPVII have multiple effects, such as anti-tumor [7,8], anti-inflammatory [9], organ protection, immune regulation, hemostasis, and analgesia [10]. Therefore, improving the quality of the *P. polyphylla* medicinal materials is of great significance for further exploring their medicinal value and providing high-quality medicinal materials for the market. *P. polyphylla* grows in the mountains and forests of Zhejiang, Yunnan, Sichuan, and other provinces of China [11]. With the exploitation of wild resources, *P. polyphylla* samples have struggled to meet the requirements of the pharmaceutical industry. In order to ensure that these requirements are met and that the quality is maintained, artificial cultivation and composition regulations have become the key approaches. As is well known, shortwave light is dominated by blue light, and violet light and UV light in forest environments have a significant impact on the growth and primary and secondary metabolism of many herbs. However, the role of shortwave light on *P. polyphylla* is not clear and the related literature is scarce. UVA and UVB commonly exist in the natural environment, and they have a clear influence on secondary metabolism. For example, Changling et al. [12] found that UVB affected *Panax notoginseng* (Burk.) F. H. in field planting, and the total content of saponins increased in a dose-dependent manner which promoted the accumulation of total saponins. Kilambi et al. [13] found that UVA and blue light improved the transcription and expression of multiple genes in the accumulation pathway of methyl-d-erythritol phosphate (MEP) and the synthesis pathway for carotene in steroidal saponin synthesis by activating photo-promoting protein in a non-phototropic seedling mutant of the tomato (*Solanum lycopersicum*). Based on *Cape gooseberry* research, Şahİn et al. [14] found that the relative expression of the squalene synthase gene was increased, respectively, by 1.34 and 2.01 times with 15 and 30 min UVB (313 nm) treatment. Although UVC is a kind of shortwave light that does not exist in the natural environment, it can regulate plants’ metabolisms. Bai et al. [15] found that appropriate doses of UV-C radiation (30 min to 3 h) induced eustress of *Tetrastigma hemsleyanum* Diels et Gilg, which contributed to the accumulation of flavonoids and improved the protective enzyme system’s activities and bioactive compound contents. Many studies have shown that UVC is absorbed by the DNA and RNA of organisms and has a certain damaging effect on cells [16,17,18]. Therefore, it is crucial to study the effect of shortwave light on the growth and saponin content of *P. polyphylla* and its related mechanism to improve the active ingredient content of *P. polyphylla*.

The purpose of this study is to investigate the effects of three types of UV light (UVA, UVB, and UVC) on *P. Polyphylla* irradiated for different periods, to explore the effects of UV light on *P. Polyphylla* plants and its saponin components (PPVI and PPVII). Three hypotheses were tested: (1) UV light treatment can regulate the level of saponins in *P. Polyphylla*; (2) there is a certain correlation between the regulatory ability of UV light on PPVI and PPVII and the activity of saponin synthase in *P. Polyphylla*; and (3) UV light treatment can cause certain oxidative damage to the plant itself, resulting in changes in MDA content and photosynthetic pigments, stimulating changes in the secondary metabolites PPVI and PPVII of the plant. Our research will improve the depth and width of research on this topic and provide a theoretical basis for industrial development.

## 2. Materials and Methods

### 2.1. Instruments and Reagents

Instruments: ACQUITY HPLC 2695 high-performance liquid chromatography (Waters, Milford, MA, USA); 250 mm C18 silica gel chromatographic column (Tianjin Wanxianghengyuan Tech Co., Ltd., Tianjin, China); 0.22 μm filter film (PTFE, Tianjin Jinteng Experimental Equipment Co., Ltd., Tianjin, China); microplate reader (BioTek Instruments, Inc., Highland Park, Winooski, VT 05404-0998, USA); and UV spectrophotometer (UNICO 3802 UV/VIS Spectrophotometer).

Reagents: PPVI and PPVII standards (Tianjin Wanxianghengyuan Technology Co. Ltd., Tianjin, China); enzyme-linked immunosorbent assay kit for Squalene synthase (SS) activity, Cycloartenol synthase (CAS), Cytochrome P450 (CYP 450) and Glycosyl Transferases (GT) (Shanghai Weilan Industrial Co., Ltd., Shanghai, China); acetonitrile (Chromatographic pure, Tedia Anhui Tiandi high purity solvent Co., Ltd., Chuzhou, China); methanol (Chromatographic pure, Sigma Anhui Tiandi high purity solvent Co., Ltd., Chuzhou, China) and other conventional reagents.

### 2.2. Treatment Method for P. polyphylla

The experiments were conducted in the Zhejiang Provincial Key Laboratory of Resource Protection and Innovative Utilization of Traditional Chinese Medicine, the State Key Laboratory of Subtropical Forest Cultivation. Seedlings of *P. polyphylla* were purchased from Lin’an Planting Base (Hangzhou City, Zhejiang Province), and identified by Dr. Xia of Zhejiang A& F University.

In October 2020, each of the 3-year-old seedlings was planted in 20 cm diameter plastic pot in Zhejiang Provincial Key Laboratory Practice Base (Lin’an, Zhejiang province, 30°15′30.39″ N, 119°43′26.92″ E). The substrate ratio was peat—pastoral soil—perlite—cow dung = 4∶4∶4∶1. Thereafter, the seedlings were routinely managed with watering, fertilizing, and weeding.

Eight months later, seedings that grew healthily and to a similar height and size were selected and treated with UV and white light. Of the pots, 9 were pre-tested for observing apparent characters, and 40 pots were randomly divided into 4 groups to determine various indexes.

### 2.3. Changes in Apparent Characters of P. polyphylla Treated by UV Light

**Figure 1:**
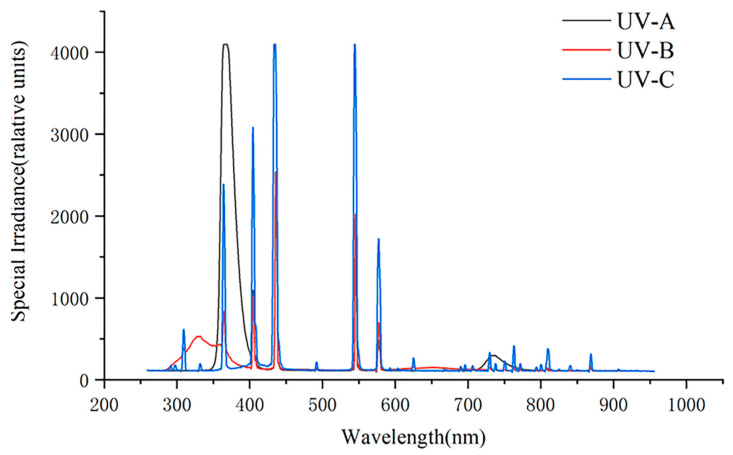
Spectrum of UVA, UVB, and UVC.

Observations and trial tests of apparent characteristics (pre-experiment): Nine seedlings were moved into the laboratory and adapted for 15 days. We installed 40 W UVA (320–400 nm, 40 W, Figure 1), UVB (280–320 nm, 40W, Figure 1), and UVC (200–280 nm, 40 W, Figure 1) on the shelf with a black cloth covering, and used them to irradiate the plants constantly. Whole plants were observed, and the changes were recorded every 15 min until there was no difference.

Formal test: Based on the apparent characteristics and corresponding times in three groups of seedlings, UV type and treatment time were defined, respectively, and recorded as UVA-90 min, UVA-4 h, etc. Seedlings treated with white light were used as the control group (CK). After light treatment, 5 pots with similar characteristics were selected from each group to determine related indexes (*n* = 5).

### 2.4. Determination of Parissaponin Content

#### 2.4.1. Conditions and Methods

The HPLC method was used to determine both PPVII [1] and PPVI [19]. An HPLC instrument and C18 chromatographic column (4.6 mm × 250 mm, 5 μm) were used. The mobile phase was acetonitrile–water. Gradient elution was carried out according to Table 1. The flow rate was 0.8 mL/min, nm, the injection volume was > 10 μL, the detection wavelength was 203 nm, and the column temperature was 30 °C.

#### 2.4.2. Sample Treatment

*P. polyphylla* sample powder: Root tubers of the sample were selected and cleaned, dried with low temperature (−40 °C) to a constant weight, smashed, and filtered through 80 mesh.

Mixed standard solution: 10.0 mg PPVI and 10.0 mg PPVII standards were weighed precisely; they were dissolved with methanol and fixed to 25 mL. We accurately measured a 2.5 mL standard solution of PPVI and PPVII into a 10 mL volumetric flask, determined its capacity with methanol, and mixed the solution well. The solution was thoroughly mixed and passed through a 0.22 μm millipore filter.

Test solution: 0.5 g of the sample powder was weighed precisely and put into a 50 mL round-bottom flask with 25 mL of absolute ethanol and weighed. A heating reflux was performed for 30 min and the mixture was cooled to room temperature. The mixture was then weighed again, and absolute ethanol was added to make up for the mass. The test solution was mixed well and filtered through a 0.22 μm millipore filter.

#### 2.4.3. Methodological Investigation

The standard curve: An investigation to test for a linear relationship was performed by using 0.5, 2.5, 5, 7.5 and 10 mL mixed standard solution and 10 mL standard solution. These were measured precisely and placed into 10 mL volumetric flasks. Their capacity was determined with methanol before being mixed. HPLC was used to determine the peak area with a 10 μL injection volume. The standard curve was drawn with the standard injection volume as abscissa (x), and the peak area was ordinate (y).

Precision test: The same sample solution was used for repeated injections and tested six times. The peak area was obtained, and the RSD was calculated.

Stability test: The same sample solution was taken and kept near to room temperature. Stability was determined after preparation for 0, 2, 4, 8, 12, and 16 h. The values of peak area and RSD were calculated.

Repeatability test: The same samples were selected and prepared as 6 flask solutions to be tested. The values of peak area and RSD were calculated.

Sample recovery test: Six samples with known PPVI and PPVII contents were weighed precisely (0.25 g for each sample) to prepare the extraction solution. The corresponding standard solution was added to the extracting solution in a 1:1 ratio. The recovery rate and RSD values were calculated.

Determination of sample content: All sample solutions were prepared according to the operations outlined in Section 2.4.2, and tested under optimized chromatographic conditions. The PPVI and PPVII contents were calculated and analyzed.

### 2.5. Determination of Activity of Synthases for Parissaponin

The enzyme-linked immunosorbent assay-double antibody sandwich method was adopted to determine the activity of SS, CAS, CYP450 (total activity), and GT (total activity) synthase. The solution was prepared according to the kit’s instructions. A Microplate reader determined the standard curve, and the synthase activity was calculated (U/L).

The principle of the enzyme-linked immunosorbent assay (ELISA) is based on the specific binding of antigens and antibodies, as well as the catalytic amplification of enzymes. Firstly, known antibodies or antigens are immobilized on a solid-phase carrier (such as a test plate) to maintain their immunological activity. Subsequently, antigens or antibodies in the sample to be detected bind specifically to these immobilized antibodies or antigens, forming antigen–antibody complexes. Then, a washing step is performed to remove unbound components, ensuring that only specifically bound antigen-antibody complexes are retained. Next, enzyme-labeled antibodies (targeting the bound antigen or antibody) are added, and these enzyme-labeled antibodies further bind to the antigen–antibody complexes, forming enzyme-labeled complexes. Finally, the substrate of the enzyme reaction is added. The enzyme will catalyze the substrate to undergo a chemical reaction, generating a detectable signal (such as color change).

### 2.6. Determination of Physiological Indices

Membrane lipid peroxidation often occurs when plant organs are aging or damaged under stress. Malondialdehyde (MDA) is the final decomposition product of membrane lipid peroxidation, and its content can reflect the degree of stress damage experienced by plants. The degree of membrane lipid peroxidation can be understood from the levels of MDA as allowing us to indirectly determine the degree of damage to the membrane system and the stress resistance of plants.

The contents of malondialdehyde and photosynthetic pigments were determined and calculated using the method proposed by Hesheng Li [20].

### 2.7. Computer Processing and Analysis of Data

To examine the differences between PPVI, PPVII, saponinase activity, MDA, and photosynthetic pigments, we analyzed these data using ANOVA with a significance level of 5% (*p* < 0.05). The normality and homogeneity of the variances were tested, and the data were logarithmically converted if necessary to reduce variance heterogeneity. ANOVA was performed using the IBM SPSS statistical software 26.0 (Armonk, NY, USA). After Z-score normalizing the average of the above data, Pearson’s analysis was performed using Origin 2021 (Northampton, MA, USA) to analyze the relationship between PPVI, PPVII and saponinase activity, photosynthetic pigments, MDA. When the *p* value < 0.05, then the two indicators are considered to be significantly correlated. Data are the average of three independent biological replicates.

## 3. Results and Discussion

### 3.1. P. polyphylla Characteristics Clearly Changed with UV Treatment

**Figure 2:**
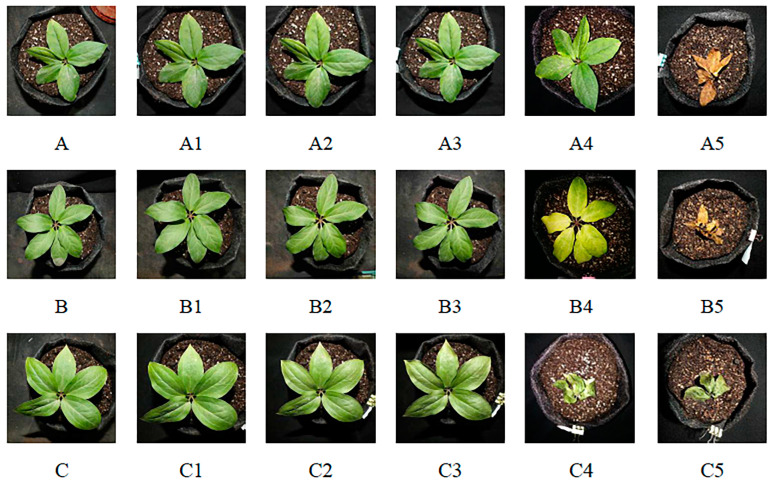
Changes in *P. polyphylla* leaves after UV treatment ((**A**–**C**): no UV treatment; (**A1**–**A3**): UVA treatment for 45 min, 90 min, 4 h; (**B1**–**B3**): UVB treatment for 30 min, 90 min, 4 h; (**C1**–**C3**): UVC treatment for 15 min, 4 h, and 6 h; (**A4**,**B4**,**C4**): UV treatment for 72 h; (**A5**,**B5**,**C5**): 7 days after UV treatment.

The apparent changes in the characteristics of the *P. polyphylla* seedlings included the process of leaf curl, yellowing or wilting, and death with an increase in UV irradiation time (Figure 2). The time it took for the types of UV to cause leaf curl differed (UVA-90 min, A2; UVB-30 min, B1; UVC-15 min, C1); after 4 h, all of the seedlings had turned yellow (A3, B3) or wilted (C3). The damage to the seedlings caused by the UV continued to worsen after about 72 h, and the plants in the UVC group died. All seedlings died after 7 days of UV treatment; interestingly, the leaves of the UVA and UVB groups were yellow, but those of the UVC group were still green.

In the pre-experiment, the changes in the leaves’ shapes and colors under different types of ultraviolet light irradiation were different. It was speculated that metabolisms should be more complex at the different UV treatment time nodes, so these time nodes were determined in the following experiment (Table 2).

### 3.2. Impact of UV Treatment Groups on the Contents of PPVI and PPVII

#### 3.2.1. Methodological Investigation Results

The standard curves of PPVI and PPVII were y = 486,178x – 17,730, y = 318,393x – 13,975. The *R*^2^ values were 0.9994 and 0.9991, which meant that the linear relationship was strong.

**Figure 3:**
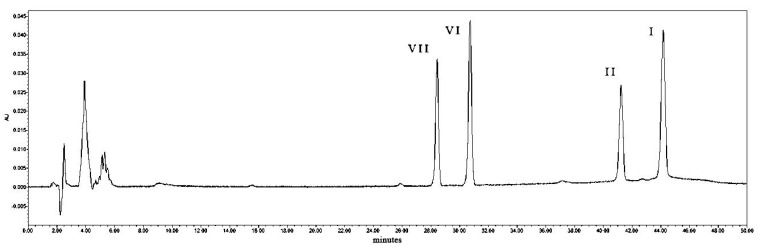
Saponin standard HPLC (VI: PPVI; VII: PPVII; I PPI; II: PPII).

An HPLC diagram of the *polyphyllin* standard is shown below (Figure 3).

In the precision test, the RSD of the peak area was in the range of 0.27–2.57%, which implied that the precision of the instrument was good. The RSD values were, respectively, 0.82% and 0.96% in the stability test, which suggested that the stability of the sample solution within 16 h was excellent. In the replication test, the RSD values were in the range of 1.45–4.73%, which meant that reproducibility was good. The recovery rates were 95.5–98.2% and the RSD values were 0.14–2.62%.

#### 3.2.2. Impact of UV Treatment on PPVI and PPVII Contents

**Figure 4:**
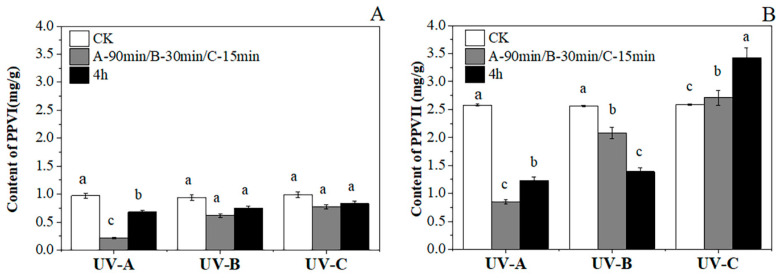
The contents of PPVI (**A**) and PPVII (**B**) in plants treated with UV light. Different lowercase letters indicate a significant difference at the 0.05 level at the same light wave condition at different irradiation times (*p* < 0.05).

As shown in the figure, the PPVI and PPVII contents of the plants in the three UV light groups were compared with those in the CK group after irradiation for different lengths of time. In the CK group, the content of PPVII (2.58 mg/g) was decidedly higher (2.66 times) than that of PPVI (0.96 mg/g); after UV treatment, the content of PPVII was 2.68 times higher than that of PPVI. In particular, the PPVII content showed a continuous upward trend in the UVC group, which increased by 32.43% within 4 h and was considered significant. The contents of PPVI and PPVII in the other five groups decreased compared with the CK. Under all UV lights, the content of PPVI decreased first and then increased, and a huge difference appeared only in the UVA group. The PPVII Content in the UVA group showed the same trend as PPVI, but in the UVB group, it explicitly showed a continuous downward trend (Figure 4).

### 3.3. Synthetase Activity Increased under UV Treatment

#### Changes in Saponin Synthase Activity

**Figure 5:**
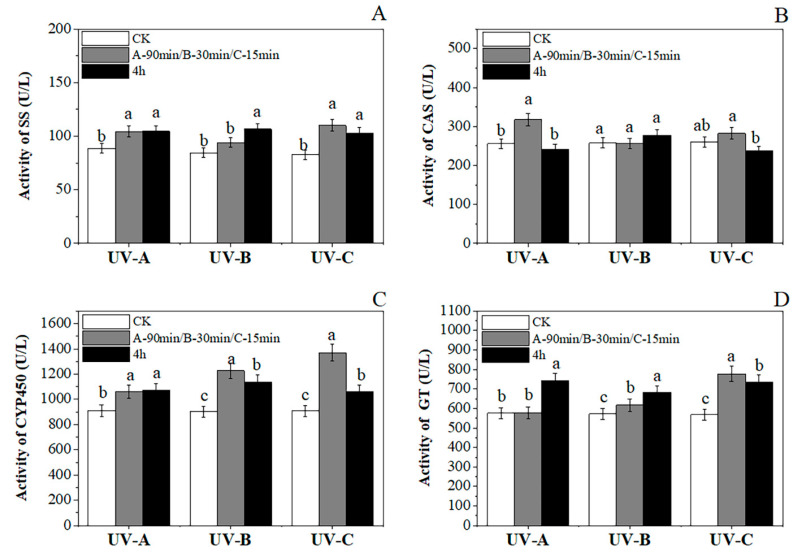
Changes in synthases activity of SS (**A**), CAS (**B**), CYP450 (**C**) and GT (**D**). Different lowercase letters indicate a significant difference at the 0.05 level at the same light wave condition at different irradiation time (*p* < 0.05).

We detected the activities of four enzymes in the UVA, UVB, and UVC groups after two time periods and compared them with their corresponding CK groups, and the differences were standardized at the 0.05 level, as shown in Figure 5.

The four synthases (SS, CAS, CYP450, GT) in the parissaponin synthesis pathway responded rapidly and showed an overall upward trend (average activity increased, respectively, by 22.93%, 10.83%, 20.15%, and 25.98%) after UV treatment (Figure 5). With UVA treatment, the CAS content increased first (24.3%) and then decreased at 4 h; the other three synthetases showed no decreasing trend. In the UVB group, the activities of SS, CYP 450, and GT explicitly increased, but CAS showed a small fluctuation only. In the UVC group, the response of CYP450 was more intense; the four synthetases showed a trend of increasing first and then decreasing. Amazingly, the GT activation experienced a more rapid and significant increase (617.30 U/L, 776.83 U/L) in a shorter time in the UVB (30 min) and UVC (15 min) groups than occurred (577.62 U/L) in the UVA group in 90 min.

### 3.4. Content of MDA Increased under UV Treatment

**Figure 6:**
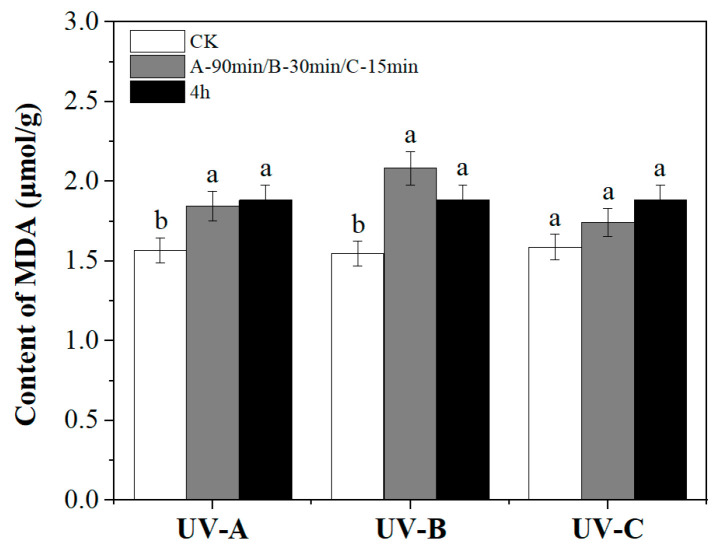
Changes in MDA content with UV treatment. Different lowercase letters indicate a significant difference at the 0.05 level at the same light wave condition at different irradiation time (*p* < 0.05).

MDA is a common oxidation product after stress in plants and causes damage to leaves. Compared with the CK, the MDA content in *P. polyphylla* increased in all UV treatment groups (Figure 6). The average MDA content in the UVA and UVB groups increased significantly by 18.82% and 28.03% compared with the CK (1.55 μmol/g), but there was no significant difference in the UVC group, although the content still increased.

### 3.5. Changes in Photosynthetic Pigment Content

**Figure 7:**
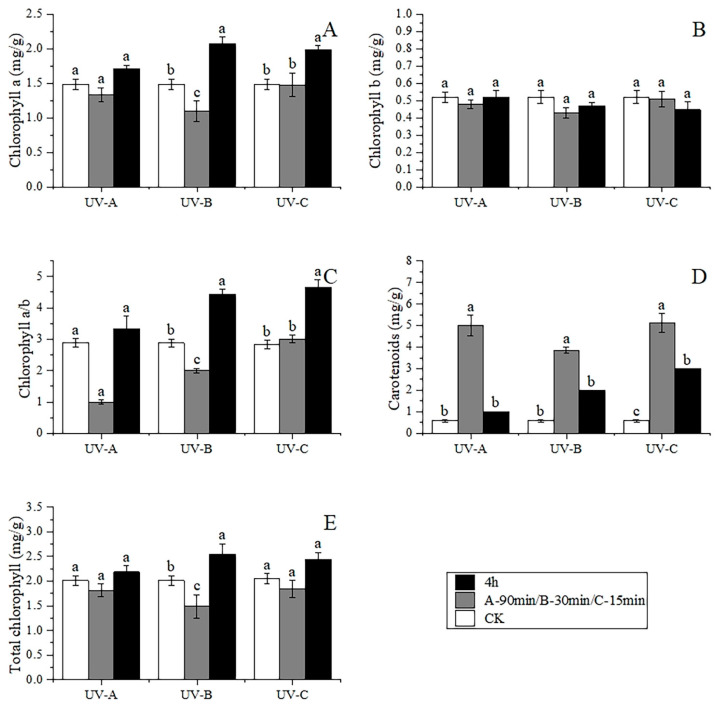
Changes in photosynthetic pigment content under UV treatment (**A**): chlorophyll a, (**B**): chlorophyll b, (**C**): chlorophyll a/b, (**D**): carotenoid), **(E**): chlorophyll a + b. Different lowercase letters indicate a significant difference at the 0.05 level at the same light wave condition at different irradiation time (*p* < 0.05).

The response of photosynthetic pigments to UV was divided into two opposing extremes (Figure 7). Carotenoid content increased rapidly in a short time after UV light treatment, by 7.58, 5.60, and 7.76 times, compared with the CK, and then experienced a cliff-like decline by 72.95%, 76.36%, and 60.14% with continuous exposure to UV light. Chlorophyll a content experience a slight fluctuation under UVA irradiation but increased by 42.56% and 35.45% within 4 h following the UVB and UVC treatments; on the contrary, there was no significant difference in the chlorophyll b content in three UV groups. Total chlorophyll (chlorophyll a + b) was consistent with chlorophyll content; chlorophyll a/b increased by 53.42% and 64.05% within 4 h with UVB and UVC light.

### 3.6. Correlation Analysis

**Figure 8:**
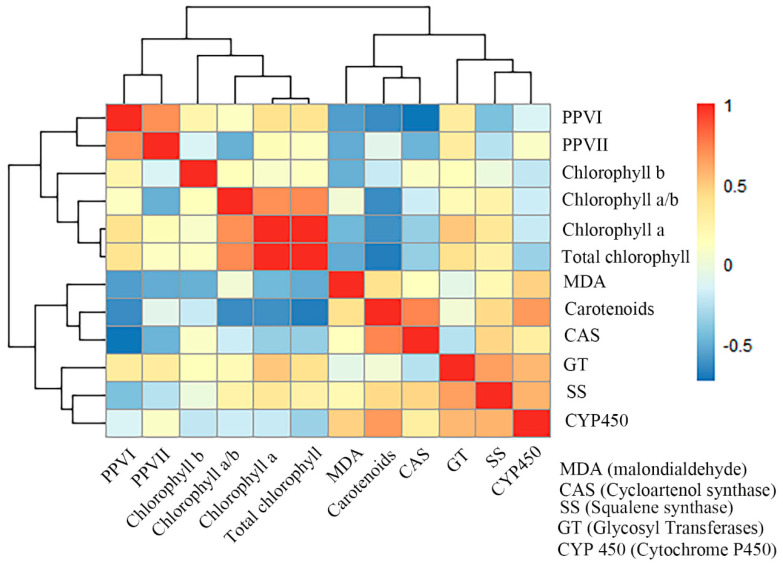
Heat map of correlation analysis of parissaponin and other substances.

The correlation analysis results showed that PPVI and PPVII had negative correlations (*p* < 0.05) with SS, CAS, MDA, and carotenoids, and they had positive correlations (*p* < 0.05) with GT and chlorophyll. PPVI showed a negative correlation (*p* < 0.05) with CYP450 and was different from PPVII. Carotenoids showed a strongly positive correlation (*p* < 0.05) with MDA, SS, CAS, GT and a significantly negative correlation (*p* < 0.05) with total chlorophyll (Figure 8).

## 4. Discussion

As a shade plant, *P. polyphylla* grows in mountain areas under forests with a degree of cover ranging from 70% to 80% [21,22]. These areas receive more shortwave light [23], which regulates saponin secondary metabolites directly or indirectly. For example, Bo et al. [24] found that blue light (455 nm) was conducive to the accumulation of parissaponin in *P. polyphylla* and promoted the content to a peak value after 30 days. Likewise, Qingtao et al. [25] found that the total biomass (whole plant dry weight) and total saponin content in *P. polyphylla* var. *yunnanensis* in the blue light (460 nm) group were significantly higher (1.98 and 1.79 times) than those in the white light groups. Lee et al. [26] also found that UV induced secondary metabolites (phenylpropanoid, phenols) and upregulated gene expression-related synthesis in a study of *Brassica napus*. These results showed that shortwave light regulated or affected saponin synthesis and are consistent with our study (PPVII content increased by 1.32 times with UVC treatment).

The synthesis of parissaponin depends on the participation of synthases, whose activity could be affected by UV. In our paper, a UV light signal rapidly activated key synthase systems (SS and CAS) in the front of the parissaponin synthesis pathway [27,28] (PSP), the synthase system (CYP450) in the middle of PSP, and the synthase system (GT) at the end of PSP. SS catalyzed isopentenyl pyrophosphate and other substances to produce oxidosqualene in the MVA (mevalonate) pathway and MEP pathway [29], and the expression of SS synthetase genes increased with UVB treatment [14]. In the following steps, mono-oxygenated squalene oxide produced 2,3-oxidosqualene with several catalytic synthases, and cycloartenol with different conformations were produced with CAS catalysis [30]. Thereafter, the CYP450 enzyme family further modified the structure of cycloartenol [31], including a hydroxylation modification of the saponin maternal nucleus [32]. GT activation was the last step in the biosynthesis pathway of parissaponin, and it was the key step in catalyzing the glycosylation of the steroidal saponin skeleton [33,34], Tang et al. [35] found that polyalthic acid in GTs is likely to endow the plant with enhanced tolerance to UV stresses. In this paper, GT was activated by UV irradiation for a certain amount of time (UVA 15 min, UVA 4 h, UVB 4 h, and UVC 15 min) and had a positive correlation with the contents of PPVI and PPVII, which differed from the response of the other three synthases. Clarifying the influence of crux GT on the syntheses of PPVI and PPVII and its mechanism should be the focus of further studies.

**Figure 9:**
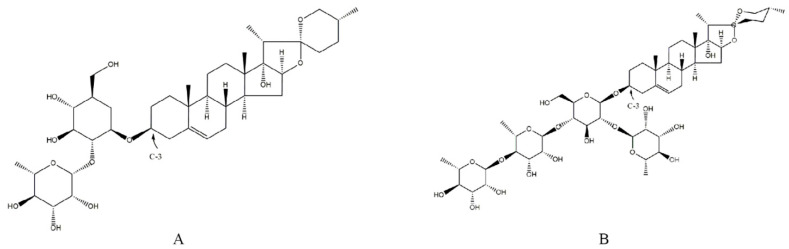
Chemical structures of PPVI and PPVII ((**A**): PPVI (CAS: 55916-51-3), (**B**): PPVII (CAS: 68124-04-9)).

However, secondary metabolism is a convoluted process and these substances are consumed under environmental stress to protect plants from oxidative damage [36]. WenChen et al. [21] studied the effect of different light intensities on *P. polyphylla* and considered that parissaponin content was significantly negatively correlated with MDA (14.3 μmol/g under 70% shading, 11.9 μmol/g under 80% shading), and found that the contents of parissaponin in 70% shading (3.79 mg/g) was lower than that in 80% shading (7.19 mg/g). Bo et al. [37] discovered that MDA content increased rapidly when water stress treatment for 2 h in *P. polyphylla*. These two studies were similar to our study in that the PPVII content increased to a certain extent under continuous UVC stress, and the plant morphology also changed rapidly (leaves depressed simultaneously within UVC 15 min). Compared with PPVII, the initial content of PPVI was lower, and the changes were more stable. Although PPVI and PPVII have the same maternal nucleus skeleton, their side chain groups are different, which might be the reason for their different antioxidant capacities. The C-3 part of PPVI is connected to rhamnose and one glucose, whereas the amount of glucose in the C-3 sugar chain of PPVII is three times that of PPVI (Figure 9). Glucose, as an aldehyde sugar, has a high reducibility [38], which means that PPVII has a stronger ability to resist oxidative stress in stressful circumstances. This factor might also be one reason for the higher basic content of PPVII in *P. polyphylla*. In summary, changes in enzyme contents and structure suggested that PPVI and PPVII may resist photooxidative damage via the plant antioxidant system. Interestingly, only PPVII content increased continuously under UVC regulation in our experiment; this phenomenon is worth studying further. The regulation of saponins by ultraviolet light has been reflected in notoginsenosides. In a field experiment on a one-year-old *Panax notoginseng* under UV-B stress, Changling et al. found that the total saponin content increased with the increase in UVB dose [12], which also boosted our confidence to continue with this in-depth study of field experiments.

PPVI and PPVII content were obviously negatively correlated with carotenoids; this meant that, while PPVI and PPVII are antioxidants, the number of carotenoids rose rapidly. The reason for this was that the carotenoids mainly absorbed shortwave light and were sensitive to shortwave light [39]. Li et al. [40] found that blue light could rapidly promote the accumulation of carotenoids. Heinze et al. [41] found that low-intensity UV irradiation increased the zeaxanthin content in plants (one kind of carotenoid). Moreover, carotenoids might be a kind of protecting pigment [42]; for example, Santin et al. [43] believed that carotenoids could protect chlorophyll from UVB photooxidative damage as they are effective scavengers of reactive oxygen species. Carotenoid content increased through the synergistic effect of multiple pathways in the later stage of treatment, which reduced the damage that the UV light inflicted on the leaves [44]. Chlorophyll was also regulated by UV light; Nwoba et al. [45] studied the impact of blue light (400–520 nm) on *Dunaliella salina* and found that chlorophyll a + b increased by 35%. This result is consistent with our study which showed that chlorophyll a + b under UVC treatment was 33% higher than that in the CK group. The chlorophyll a/b values of the three groups were higher than those of the CK group.

## 5. Conclusions

The results of this study indicate that UV light can induce the stress response of *P. polyphylla;* the synthesis enzyme nodes (GTs) in the saponin synthesis pathway were activated, thereby promoting the accumulation of saponin components. The plant responds to UV light damage by depleting carotenoids and secondary metabolite saponins, stimulating the activity of related saponin synthase and further regulating the synthesis and accumulation of saponins. The data showed that carotenoids decrease rapidly and the MDA content increases after UV irradiation, indicating that the plant is under UV stress. UVA exhibits a faster response in the form of increasing PPVI and PPVII contents than UVB, with a significant increase observed after 4 h of treatment. However, UVB does not significantly affect PPVI content, while PPVII content significantly decreases. It is worth noting that although UVC treatment does not significantly affect PPVI content, PPVII content increases significantly with continuous exposure to UVC. We speculate that the different responses of PPVI and PPVII to UV may be related to their structure, as PPVII exhibits stronger resistance to stress damage, which warrants further research. Furthermore, the impact of UV light irradiation on gene-level changes and the underlying mechanism still require further exploration. Additionally, UV irradiation positively affects the activity of saponin synthase, activating GTs.

In summary, the results of this study provide a theoretical basis and new insights for improving saponin content in *P. polyphylla* through UV light regulation. In our subsequent research, we will scientifically refine and allocate the radiation time and plant repair time based on the type of UV type and the length of exposure (UVA-4h, UVC) that can significantly improve the levels of PPVI and PPVII found in this study. We will also carry out further research and analyses to explore the internal mechanism of UV light radiation which affects saponin accumulation.

## Figures and Tables

**Table 1:** Gradient elution program of the mobile phase.

Time (min)	Acetonitrile	Water (%)
0	30	70
40	60	40
50	30	70
90	30	70

**Table 2:** Periods of subjecting *P. polyphylla* to different UV light treatments.

UV Light	Treatment	Record
UVA	90 min	UVA-90 min
	4 h	UVA-4 h
UVB	30 min	UVB-30 min
	4 h	UVB-4 h
UVC	15 min	UVC-15 min
	4 h	UVC-4 h

## Data Availability

The raw data supporting the conclusions of this article will be made available by the authors on request.
